# Prolonged efficacy of the 300IR 5-grass pollen tablet up to 2 years after treatment cessation, as measured by a recommended daily combined score

**DOI:** 10.1186/s13601-015-0057-8

**Published:** 2015-05-22

**Authors:** Alain Didier, Hans-Jørgen Malling, Margitta Worm, Friedrich Horak, Gordon L Sussman

**Affiliations:** Rangueil-Larrey Hospital, Department of Respiratory Diseases, 24 Chemin de Pouvourville, TSA 30030 31059 Toulouse, Cedex 9 France; Allergy Clinic, Copenhagen University Hospital, Gentofte, Denmark; Department of Dermatology and Allergy, Charité – Universitätsmedizin Berlin, Berlin, Germany; Vienna Challenge Chamber, Institute for Allergy Research, Vienna, Austria; Division of Allergy and Clinical Immunology, University of Toronto, Toronto, ON Canada

**Keywords:** 5-grass pollen tablet, Allergen-specific immunotherapy, Allergic rhinitis, Allergic rhinoconjunctivitis, Long-term efficacy Sublingual immunotherapy

## Abstract

**Background:**

The 300IR (index of reactivity) 5-grass pollen tablet has favorable short-term and sustained clinical efficacy in patients with grass pollen-induced allergic rhinoconjunctivitis (ARC). Here, we report maintenance of efficacy and safety over 2 years following treatment discontinuation.

**Methods:**

Randomized, double-blind, placebo-controlled, parallel-group, multicenter Phase 3 trial in patients aged 18–50 years with ARC. During study years 1–3, patients received a daily sublingual tablet containing either 300IR 5-grass pollen extract or placebo, according to a discontinuous pre- and coseasonal protocol. Study years 4 and 5 were treatment-free. In response to health authorities’ recommendations, the daily combined score (DCS) was assessed in a *post-hoc* analysis as the efficacy endpoint. Components of the DCS were daily rhinoconjunctivitis total symptom score (DRTSS) and daily rescue medication score (DRMS).

**Results:**

633 patients with ARC were randomized to placebo (n = 219) or 300IR 5-grass pollen tablet, beginning 4 months (4 M, n = 207) or 2 months (2 M, n = 207) prior to the estimated start of the grass pollen season and continuing until season’s end. During the first post-treatment year, a statistically significant difference versus placebo in least squares (LS) mean DCS was noted in patients previously receiving active treatment (300IR (2 M) point estimate: −0.16, 95% confidence interval (CI_95%_): [−0.26, −0.06], p = 0.0019; −31.1%; 300IR (4 M) point estimate: −0.13, CI_95%_: [−0.23, −0.03], p = 0.0103, −25.3%). During the second post-treatment year, patients in the 300IR (4 M) group, but not the 300IR (2 M) group, showed a statistically significant difference in LS mean DCS versus placebo (point estimate: −0.11, CI_95%_: [−0.21; 0.00], p = 0.0478, −28.1%). This significant efficacy seen during the post-treatment years in patients previously treated with 5-grass pollen tablet compared favorably with that during the 3 prior years of active treatment. A statistically significant difference versus placebo was also noted in secondary efficacy measures in both post-treatment years (except for DRTSS in year 5). In the absence of any active treatment, the safety profile was similar in the active groups versus placebo group during either post-treatment year.

**Conclusions:**

In adults with grass pollen-associated ARC, 5-grass pollen tablet therapy beginning 4 months before the pollen season and continuing to season’s end demonstrated efficacy across all variables during active treatment, and this effect was prolonged for up to 2 years post-treatment.

**Trial registration:**

ClinicalTrials.gov identifier: NCT00418379.

## Background

Allergic rhinitis (AR) affects an estimated 500 million people globally, and is increasing in prevalence in many countries [1]. AR places a heavy burden on healthcare resources, and is associated with substantial indirect costs related to absenteeism from work and decreased productivity [2].

The 300IR (index of reactivity) 5-grass pollen tablet (Oralair®; Stallergenes, Antony, France) is approved as allergen immunotherapy (AIT) for the treatment of confirmed grass pollen-induced AR with or without conjunctivitis in over 30 countries worldwide and is the first registered AIT tablet in the United States. Its short-term clinical efficacy has been well-established through a large number of studies conducted in adults and children, which demonstrated consistent results [3-5]. The 5-grass pollen tablet is effective from the first pollen season in controlling the symptoms of allergic rhinoconjunctivitis (ARC) and reducing the need for symptomatic medication in all types of patients, irrespective of mono- or polysensitization status, symptom severity and the presence or absence of co-morbid mild asthma [6,7].

In clinical trials, the efficacy of immunotherapy for ARC was often evaluated using the average rhinoconjunctivitis total symptom score (ARTSS) [8]. For ethical reasons, patients are provided with rescue medications, which may alleviate symptoms. Their use can result in a corresponding reduction in symptom scores, which may decrease the mean difference between the active-treatment and placebo groups [8]. Based on this rationale, World Allergy Organization (WAO) taskforce recommendations on the methodological aspects of immunotherapy clinical trials propose that a combined daily symptom and rescue medication score be used as the primary outcome measure [9]. Similarly, the European Academy of Allergy and Clinical Immunology (EAACI) Immunotherapy Interest Group also recommends the use of a homogenous combined symptom and medication score as a standardized method to balance both symptoms and the need for antiallergic medication in an equally weighted manner [10]. Such a standardized combined score can provide a simple analysis of the daily burden of disease [10]. Combining symptom and medication scores with equal importance is also associated with a large effect size, which is powered to demonstrate treatment efficacy [10].

The European Medicines Agency (EMA) guidance on development of products for allergen immunotherapy states that a long-term clinical study is necessary to demonstrate maintenance of efficacy over 3 treatment years, and a disease-modifying effect after treatment discontinuation, both of which are key goals of specific AIT and distinguish it from other allergy treatments [11]. It has previously been shown that the efficacy of the 300IR 5-grass pollen tablet is sustained when administered discontinuously as a pre- and coseasonal treatment (with a treatment-free period for the other months of the year, starting after the season ends), over 3 consecutive years in adults with moderate to severe ARC (study VO53.06) [12]. Furthermore, this favorable efficacy persists for at least one season after treatment is discontinued [13]. Here, we report long-term efficacy and safety results from study VO53.06 during the first (year 4) and second (year 5) seasons following treatment discontinuation, using the daily combined score (DCS).

## Methods

### Study design

The design and detailed results from the first 4 years of this randomized, double-blind, placebo-controlled, parallel-group, multicenter, Phase 3 trial conducted at 48 sites in Austria, Canada, Czech Republic, Denmark, France, Germany, Italy, Poland, Russia and Slovakia, have been reported previously [12,13]. The trial originally included 3 years of active treatment and 1 year of treatment-free follow-up to investigate the long-term efficacy of the 300IR 5-grass pollen tablet. At the end of year 3, the Data Safety Monitoring Board recommended that the study be extended for an additional year of treatment-free follow-up (year 5).

In brief, during the first 3 years of the study (2007–09), patients were randomized 1:1:1 using a computer-generated list to either placebo or 300IR 5-grass pollen tablet, administered according to a discontinuous pre- and coseasonal protocol beginning at 4 or 2 months (300IR (4 M) and 300IR (2 M), respectively) prior to the expected start of the grass pollen season and maintained until season’s end. Patients did not receive investigational product during years 4 and 5 of the study (2010–11). Patients and investigators remained blinded until the end of the fifth year of the study.

The study protocol (European Union Drug Regulating Authorities Clinical Trials (EudraCT) number 2006-003258-98) was reviewed and approved by local regulatory authorities and independent ethics committees in each country, and the study and year 5 extension were conducted according to the Declaration of Helsinki and Good Clinical Practice-International Conference on Harmonisation guidelines. Patients provided their written informed consent before the start of any study-associated procedure, and reconsented prior to participation in the year 5 extension.

### Patients

Patients were aged 18–50 years with documented grass pollen-related ARC for at least the two previous grass pollen seasons, a positive skin-prick test (wheal diameter >3 mm) to 5-grass-pollen-mix extract, *Phleum pratense* (timothy grass)-specific serum IgE ≥0.7 kU/L, and a retrospective rhinoconjunctivitis total symptom score (RRTSS) ≥12 (on a 0–18 scale), based on the most severe days in the grass pollen season preceding enrolment.

Exclusion criteria were ARC symptoms during the grass pollen season due to allergens other than grass pollen, asthma requiring treatment with more than a beta-2 agonist, or presence of any other disease that might affect the participation or outcome of the study.

### Treatment

During the first 3 years of the study, patients received a daily sublingual tablet containing either 300IR 5-grass pollen extract or placebo, according to the discontinuous dosing schedule described above. Years 4 and 5 of the study (i.e., the fourth and fifth pollen season) were 300IR 5-grass pollen tablet treatment-free.

To maintain blinding, active and placebo tablets were identical in appearance and taste, and patients randomized to the 2 M dosing regimen received placebo for the 2 months prior to starting active treatment.

The use of oral antihistamines, antihistamine eye drops, nasal corticosteroids and oral corticosteroids as rescue medications was permitted throughout the study, following a stepwise regimen defined in the study protocol, in which oral corticosteroids could only be considered once the other types of medications had proven inadequate at managing severe or intolerable ARC symptoms.

### Grass pollen season

During each pollen season, pollen counts were monitored and recorded daily in the regions where study sites were located. The pollen period for statistical analysis was defined as starting on the first of 3 consecutive days with a grass pollen count of ≥30 grains/m^3^ and ending on the last of 3 consecutive days with a grass pollen count of ≥30 grains/m^3^.

### Outcome measures

The main outcome measures have been reported previously [12,13]. From approximately 3 weeks before each pollen season until the end of that season, patients recorded in a daily diary six individual rhinoconjunctivitis symptom scores (RSSs: sneezing, rhinorrhea, nasal pruritus, nasal congestion, ocular pruritus and watery eyes) and use of rescue medication during the previous 24 h. Severity of symptoms was rated using a 4-point descriptor scale from 0 (absent) to 3 (severe), and the sum of all six individual RSSs comprised the rhinoconjunctivitis total symptom score (RTSS, range 0–18) [14]. The rescue medication score (RMS, range 0–3) was calculated using the following scale: 0, no rescue medication; 1, use of antihistamine (oral and/or eye drops); 2, use of nasal corticosteroid; and 3, use of oral corticosteroid. If a patient took two or more rescue medications on the same day, the highest score was used for the RMS [14]. Efficacy analyses were performed on the full analysis set (FAS). In contrast to other symptoms scores, no last observation carried forward data adjustment is carried out with the Average adjusted symptom score (AAdSS), thereby reducing the potential for skewing of the data.

After the study had commenced, guidance from health authorities and professional societies regarding the primary efficacy endpoint of AIT clinical trials subsequently recommended the use of a score that considers both symptoms and rescue medication use, to avoid an underestimation of symptoms severity. In response to this guidance, the average adjusted symptom score (AAdSS) [14] was introduced as primary efficacy measure [12,13]. Results for the AAdSS have been reported previously [12,13].

The AAdSS was calculated per patient as the average of the non-missing daily adjusted symptom scores (AdSSs, range 0–18), which were derived from the daily RTSS (DRTSS) adjusted for rescue medication use over 2 consecutive days [14].

Secondary efficacy measures included individual RSSs; Average Combined Score (ACS, range 0–3); DRTSS; daily RMS (DRMS); ARTSS (range 0–18); and average RMS (range 0–3). All average scores were patient-specific and calculated as the average of the non-missing daily scores during the pollen season while the patient was on treatment [14]. In response to regulatory authorities recommending use of a combined symptom and rescue medication score [9], a *post-hoc* analysis was performed using an alternative measure for the evaluation of efficacy: DCS (range 0–3), which assigns equivalent weighting to both symptom and rescue medication scores, and is calculated as [(DRTSS/6) + DRMS)]/2 [8,14]. DCS is a genuine combined score, unlike AdSS, which is not a combined score *per se*, but a symptom score that is adjusted by taking into account the use of allergy rescue medication [15].

In this paper, results using the alternative efficacy measure, DCS, are presented.

### Safety

Safety was assessed through monitoring of adverse events (AEs), laboratory parameters, physical examination and vital signs. AEs were recorded throughout the study and classified according to the Medical Dictionary for Regulatory Activities (MedDRA; version 9.1).

### Sample size

The results of a previous pivotal, dose-finding study in Europe of the 5-grass pollen tablet for the treatment of ARC [4] suggested that a sample size of 144 patients per group would have 80% power to detect a difference of 20% between the active-treatment and placebo groups in AAdSS during the third on-treatment pollen season (primary endpoint), given a type I error of 5% and a common standard deviation (SD) of 3.6.

For post-treatment, long-term efficacy assessment, assuming a screening failure rate of 12% each treatment year and a comparable 12% drop-out rate for the fourth year, 127 patients per group would have 75% power to detect a 20% difference in AAdSS for post-treatment long-term efficacy. No formal pre-defined power calculation was performed for the analysis of year 4 and year 5.

Statistical analyses were performed using SAS® software (version 9.1.3, SAS Institute Inc., Cary, NC, USA). The safety sets in year 4 and year 5 included all patients who received ≥1 dose of 300IR 5-grass pollen tablet during years 1–3, and who completed the first visit in year 4 (visit 19) or year 5 (visit 23), respectively. The FAS for years 1–3 included all patients who started treatment and had ≥1 AdSS recorded during the pollen season of that year. The FAS for years 4 and 5 included all patients who started the respective post-treatment year and had ≥1 AdSS recorded during the pollen season of that year. The per-protocol sets included all FAS patients with ≥14 valid AdSS days during the pollen season or valid AdSS days for ≥50% of the pollen period, and who completed the study without any major protocol deviations.

Average scores were analyzed using a linear model and an analysis of covariance (ANCOVA) with pooled site, age, gender, asthma status and sensitization status as covariates. The *post-hoc* analysis of the DCS was performed using a linear mixed model with repeated measures and an ANCOVA. Variables which could potentially impact the clinical score (pooled site, age, gender, asthma status and sensitization status) were included in the model as covariates. Treatment was the main effect and the day was the indicator of time. All effects were fixed, except the patient who was considered as a random effect. A point estimate and 95% confidence interval (CI_95%_) for the difference in adjusted means between active-treatment and placebo groups were calculated. The threshold for statistical significance was set at p ≤ 0.05 and all inferential tests were two-sided.

DRTSS and DRMS, the components of the DCS, were analyzed similarly to the DCS.

## Results

### Study population

Demographics and baseline characteristics for the FAS_Year 4_ have already been published [13]. Data from FAS_Year 5_ were well-balanced and comparable across treatment groups (Table 1). Randomized patients in the FAS_Year 5_ had moderate-to-severe grass pollen-associated ARC, as denoted by a mean RRTSS of 14.1 (SD: 1.7), for a mean duration of 12.8 years (SD: 9.0). The majority (59.4%) of patients were polysensitized to grass pollen and another allergen, and Global Initiative for Asthma (GINA) treatment Step I for asthma [16] was reported in 12.7% of patients.

**Table 1:** **Demographics and baseline characteristics of the study population (FAS**_**Year 5**_ **, N = 377)**

**Parameter**	**Treatment group**			
	**Placebo n = 133**	**300IR (2 M) n = 117**	**300IR (4 M) n = 127**	**Total N = 377**
**Gender, n (%)**				
Male	79 (59.4)	71 (60.7)	81 (63.8)	231 (61.3)
Female	54 (40.6)	46 (39.3)	46 (36.2)	146 (38.7)
**Age, years**				
n	133	117	127	377
Mean (SD)	30.1 (8.7)	30.9 (7.8)	30.5 (8.5)	30.5 (8.3)
Range	18–49	18–51	18–49	18–51
**Body mass index, kg/m** ^**2**^				
n	132	117	127	376
Mean (SD)	23.9 (4.3)	24.3 (3.9)	24.2 (3.7)	24.1 (4.0)
Range	15.6–42.4	17.3–35.1	17.2–35.1	15.6–42.4
**Duration of ARC, years**				
n	133	117	127	377
Mean (SD)	12.5 (9.0)	12.9 (8.8)	13.0 (9.2)	12.8 (9.0)
Range	1.6–44.0	1.5–39.0	1.5–42.1	1.5–44.0
**RRTSS**				
n	133	117	127	377
Mean (SD)	14.0 (1.7)	13.9 (1.8)	14.2 (1.7)	14.1 (1.7)
Range	12–18	12–18	12–18	12–18
**Asthma status, n (%)**				
Yes	19 (14.3)	13 (11.1)	16 (12.6)	48 (12.7)
No	114 (85.7)	104 (88.9)	111 (87.4)	329 (87.3)
**Sensitization status, n (%)**				
Monosensitized	53 (39.8)	47 (40.2)	53 (41.7)	153 (40.6)
Polysensitized	80 (60.2)	70 (59.8)	74 (58.3)	224 (59.4)

2 M, 2-month preseasonal dosing protocol; 4 M, 4-month preseasonal dosing protocol; ARC, allergic rhinoconjunctivitis; FAS, full analysis set; IR, index of reactivity; RRTSS, retrospective rhinoconjunctivitis total symptom score; SD, standard deviation.

**Figure 1:**
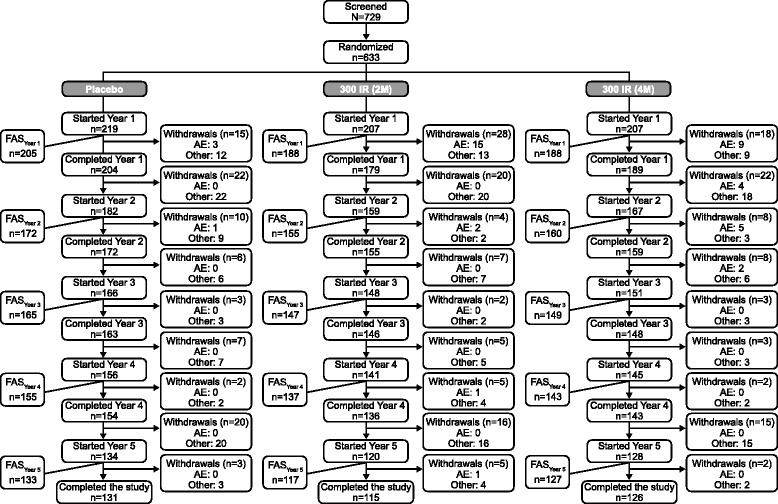
**Flow-chart of patients per treatment group and treatment season throughout the three pre- and coseasonal treatment periods (years 1–3) and post-treatment period (years 4 and 5).** 2 M, 2-month preseasonal dosing protocol; 4 M, 4-month preseasonal dosing protocol; FAS, full analysis set; IR, index of reactivity.

Overall, 633 patients with ARC were randomized to receive placebo (n = 219) or 300IR 5-grass pollen tablet starting 4 months (4 M; n = 207) or 2 months (2 M; n = 207) before the estimated start of the grass pollen season (Figure 1). Of the 457 patients who completed year 3, 15 patients withdrew from the study before the start of year 4 [placebo: consent withdrawn (n = 5), protocol non-compliance (n = 1), other reason (n = 1); 300IR (2 M): consent withdrawn (n = 3), pregnancy (n = 1), protocol non-compliance (n = 1); 300IR (4 M): consent withdrawn (n = 1), ineligibility (n = 2)]. The year 4 safety set included 442 patients (placebo, n = 156; 300IR (2 M), n = 141; 300IR (4 M), n = 145), with the FAS_Year 4_ comprising 435 patients (placebo, n = 155; 300IR (2 M), n = 137; 300IR (4 M), n = 143).

Of the 433 patients who completed year 4 (post-treatment year 1), 51 withdrew from the study before the start of year 5, with the majority citing a wish not to participate [placebo: consent withdrawn (n = 12), lack of efficacy requiring another treatment to be prescribed (n = 1), other reason (n = 7); 300IR (2 M): consent withdrawn (n = 9), lost to follow-up (n = 1), other reason (n = 6); 300IR (4 M): consent withdrawn (n = 10), planning to conceive (n = 1), other reason (n = 4)]. At the end of year 5, the safety set included 382 patients (placebo, n = 134; 300IR (2 M), n = 120; 300IR (4 M), n = 128), with the FAS_Year 5_ comprising 377 patients (placebo, n = 133; 300IR (2 M), n = 117; 300IR (4 M), n = 127). This represents a loss of 9.1% (n = 58) of patients from the end of year 4 of the study, and 40.4% (n = 256) of patients, compared with the initial year 1 population.

Notably, those patients who withdrew from the study between year 4 and the end of year 5 had markedly more severe ARC symptoms than those who continued, as measured by ARTSS. Mean ARTSS scores (± SD) for the patients who discontinued after year 4 were 5.68 ± 3.37 (placebo, n = 22), 3.15 ± 3.11 (300IR (2 M), n = 20) and 5.01 ± 4.74 (300IR (4 M), n = 16), compared with 4.04 ± 3.21 (placebo, n = 133), 3.22 ± 3.12 (300IR (2 M), n = 117) and 3.30 ± 3.19 (300IR (4 M), n = 127) for those patients who continued into year 5. Moreover, irrespective of study year, among patients who discontinued, those in the placebo group experienced more severe ARC symptoms than their counterparts in the 300IR (4 M) group. Similar differences in ARC symptom severity between patients receiving placebo or active treatment were found using ACS. Patients in the placebo group who discontinued before year 5 had a higher ACS (0.79) than those in the active-treatment groups who withdrew from the study at this time (0.49 and 0.70 for 300IR (2 M) and 300IR (4 M), respectively).

### Efficacy

**Figure 2:**
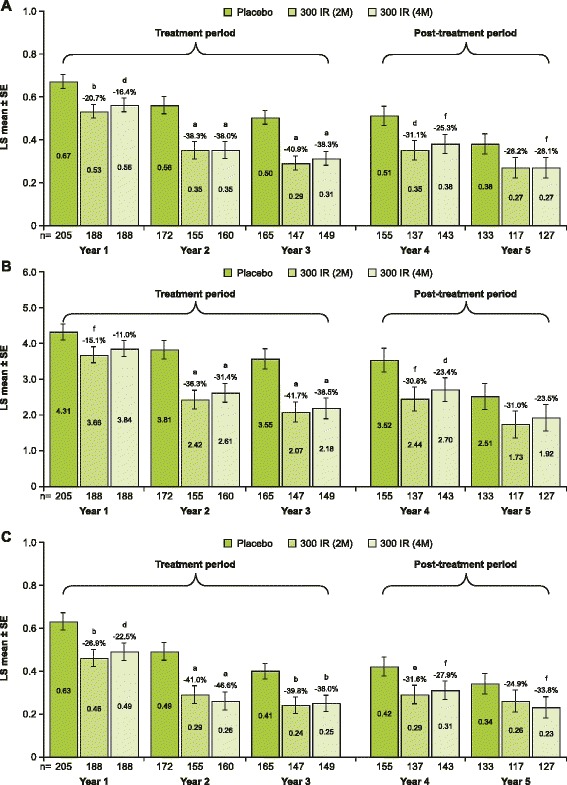
**Repeated measures analysis of covariance of the DCS (A), DRTSS (B) and DRMS (C) per year of the study (FAS**_**Year 1–5**_ **).** ^a^p < 0.0001, ^b^p < 0.0005, ^c^p < 0.001, ^d^p < 0.005, ^e^p < 0.01, ^f^p < 0.05 for comparison *vs* placebo. DCS, daily combined score; DRMS, daily rescue medication score; DRTSS, daily rhinoconjunctivitis total symptom score; FAS, full analysis set; IR, index of reactivity; LS, least squares; SE, standard error.

A statistically significant difference versus placebo in least squares (LS) mean DCS was noted in patients in the active-treatment groups during the first post-treatment year (FAS_Year 4_) (300IR (2 M) point estimate: −0.16, CI_95%_: [−0.26, −0.06], p = 0.0019, −31.1%; 300IR (4 M) point estimate: −0.13, CI_95%_: [−0.23, −0.03], p = 0.0103, −25.3%) (Figure 2A). During the second post-treatment year (FAS_Year 5_), patients in the 300IR (4 M) group, but not the 300IR (2 M) group, showed a statistically significant difference in LS mean DCS, compared with placebo (point estimate: −0.11, CI_95%_: [−0.21; 0.00], p = 0.0478), which corresponds to a relative LS mean difference of −28.1%. This compares favorably with the significant efficacy of the 5-grass pollen tablet after 3 pollen seasons of discontinuous pre- and coseasonal treatment (FAS_Year 3_), during which both the 300IR (4 M) and 300IR (2 M) groups showed a statistically significant difference in DCS, compared with placebo (p < 0.0001) (Figure 2A).

As was observed for DCS, a similar statistically significant difference versus placebo was noted in all other efficacy variables after years 4 and 5, with the exception of DRTSS (both 300IR (2 M) and (4 M) groups) (Figure 2B), AAdSS (300IR (4 M)) and ARTSS in year 5 (300IR (2 M)). Scores after treatment cessation still favored the active-treatment groups, as during the active-treatment period. The DRMS remained stable with the transition from active treatment into the treatment-free years 4 and 5 (Figure 2C).

Of note, DCS, DRTSS and DRMS were similar from years 2 to 5 in the active-treatment groups, whereas they decreased consistently in the placebo group (Figure 2). This trend for active-treatment values and steadily decreasing placebo values over the course of the study was also observed for LS mean DRTSS (300IR (2 M): 3.66, 2.42, 2.07, 2.44, 1.73 and 300IR (4 M): 3.84, 2.61, 2.18, 2.70, 1.92 versus placebo: 4.31, 3.81, 3.55, 3.52, 2.51 for FAS_Year 1–5_, respectively) and LS mean DRMS (300IR (2 M): 0.46, 0.29, 0.24, 0.29, 0.26 and 300IR (4 M): 0.49, 0.26, 0.25, 0.31, 0.23 versus placebo: 0.63, 0.49, 0.41, 0.42, 0.34 for FAS_Year 1–5_, respectively). For the comparison of DRTSS at year 4 (FAS_Year 4_) and year 5 (FAS_Year 5_), this trend was particularly pronounced, with the difference in LS mean scores being −0.71 and −0.78 for the 300IR (2 M) and 300IR (4 M) groups, respectively, versus −1.01 for placebo.

It is important to note that the difference versus placebo for the secondary criterion, ACS, was statistically significant at year 5 (FAS_Year 5_) for the 300IR (4 M) group (point estimate: −0.11, CI_95%_: [−0.21, −0.00], p = 0.0473, −20.7%), but not for the 300 IR (2 M) group (point estimate: −0.11, CI_95%_: [−0.22, 0.00], p = 0.0525, −20.7%).

### Safety

Safety profiles in the treatment-free years 4 and 5 were consistent between all groups of patients who previously received placebo or active treatment, with no unexpected signal identified during either post-treatment year. There were no reports of autoimmune disorders judged to be related to study treatment, and no notable differences regarding laboratory results, vital signs or physical examinations between treatment groups during the post-treatment period. One post-treatment AE of oral allergy syndrome occurring in year 4 in a patient previously assigned to therapy with the 300IR (4 M) regimen was judged to be related to the 300IR 5-grass pollen tablet.

## Discussion

In the first and second post-treatment years of this randomized, double-blind, placebo-controlled, multicenter trial, a statistically significant and clinically relevant difference was observed between the 300IR (4 M) group and placebo for the DCS, DRTSS and DRMS (relative LS mean differences: first post-treatment year: −25.3%, −23.4% and −27.9% respectively; second post-treatment year: −28.1%, −23.5% and −33.8%, respectively). These findings confirm that the favorable long-term ARC symptom management of the 300IR 5-grass pollen tablet extends to 2 years post-treatment, building on the results of previous analyses of this study [12,13]. When considering absolute values across the study, the scores for patients in the active-treatment groups remained relatively static between years 3 to 5, in contrast to scores from patients in the placebo group, which steadily decreased due to premature withdrawal of patients with severe ARC symptoms.

This high rate of withdrawals of the most symptomatic patients, particularly those randomized to placebo, is an inherent drawback of long-term studies. The present study was initially conducted to assess post-treatment efficacy over three treatment periods and a fourth subsequent immunotherapy-free year. The study was later extended to include a fifth immunotherapy-free year. This extension was accompanied by the withdrawal of 51 patients prior to the start of the final year. It is assumed that patients with the most severe ARC symptoms in the placebo group would have a higher chance of discontinuing before the end of the study, and this loss of patients from the placebo group can artificially reduce the difference in estimated treatment effect and introduce bias into the data. Nevertheless, significant post-treatment efficacy was noted in patients in the 300IR (4 M) group, as judged by DCS, which is the approved treatment scheme. In the second post-treatment year, the difference versus placebo in DCS for the 300IR (2 M) group was not statistically significant, but reached the same level (0.27) as the 300IR (4 M) group. Although a trend towards improved efficacy was observed in the 300IR (2 M) group, the lack of statistical significance versus placebo may be due to the lower number of patients in this group, compared with the 300IR (4 M) group. However, no firm conclusion can be drawn.

A consistent yearly decrease in patients with the most severe ARC symptoms, receiving placebo, was also observed in a comparable 5-year study in patients with grass pollen-induced ARC, which investigated the long-term efficacy of continuous treatment with a 1-grass pollen tablet preparation [17]. Significant efficacy was confirmed in this study during both the on-treatment and subsequent post-treatment years, using a daily combined symptom medication score that was weighted towards the use of rescue medication. However, in contrast to the present study, in which the use of rescue medication remained fairly constant throughout all study years, there was a marked increase in daily rescue medication use in the 1-grass pollen tablet study during the post-treatment period, compared with the preceding years on active treatment [17,18].

In the original design of the present study, the AAdSS was pre-specified as the primary efficacy measure. Of note, in year 5, AAdSS scores did not reach statistical difference for the 300IR (4 M) group, while they did for the 300IR (2 M) group, compared with placebo. The AAdSS is a symptom score that is adjusted according to allergy rescue medication use [15], which was developed by Stallergenes. However, while accepted by European health authorities, it is not a true combined scoring instrument like the DCS [15]. Weaknesses of the AAdSS are that it does not account for the magnitude of the effect of different classes of rescue medication, and the duration of adjustment is set to 2 days as a conservative approach, leading to an overestimation of rescue medication use.

Health authorities, and the WAO and EAACI, have proposed that a combined symptom and rescue medication score, in particular one assigning equivalent importance of the two components, is utilized in ARC trials [9,10]. Combined scores are comprehensive efficacy endpoints that also offer a number of benefits, including simplicity and ease of application [14]. Daily scores are either recorded by patients on a daily basis or are derived daily, whereby each day is a separate time point [10]. The lack of a standardized scoring method for assessing DCS is a recognized issue in AIT research and several different approaches have been used in the field, in addition to the one employed in this study [15]. Of note, differences versus placebo in the ACS, planned in the protocol as a secondary criterion, were also statistically significant for both active treatment groups, with the exception of 300IR (2 M) in the second post-treatment year (mirroring the results for DCS).

Although few studies have investigated the optimal duration of AIT, current recommendations advise treating patients for 3–5 years [11,19-21]. In line with these recommendations, the study reported here investigated long-term, post-treatment efficacy of the 300IR 5-grass pollen tablet when given pre- and coseasonally for 3 consecutive years, and monitored efficacy for the following 2 years. It may also be valuable to investigate extending the duration of treatment beyond 3 years, or to individualize the duration of immunotherapy.

As would be expected in an untreated patient population, there were no safety signals during post-treatment years.

The 300IR 5-grass pollen tablet offers a number of benefits. This unique pre- and coseasonal immunotherapy is the first oral treatment with a consistent, well-balanced allergen extract that mimics natural exposure and sensitization [22], and over 5 years of clinical and real-world experience has demonstrated its efficacy and safety as a therapy for grass pollen allergy [23]. Results of the present analysis add to the existing body of evidence, demonstrating that the efficacy in terms of ARC symptom control over 3 years of treatment is present for 2 years after stopping treatment.

## Conclusions

This is the first long-term study of discontinuous treatment with the 300IR 5-grass pollen tablet that indicates efficacy for up to 2 years post-treatment. Consistent *post-hoc* analysis results with the 300IR 5-grass pollen tablet beginning 4 months before the pollen season and continuing to season’s end were achieved across all efficacy variables, and stabilization of ARC symptoms was attained during both active treatment, and in the 2 subsequent years without treatment.

## Abbreviations

AAdSS: Average adjusted symptom score
ACS: Average Combined Score
AdSS: Adjusted symptom score
AE: Adverse event
AIT: Allergen immunotherapy
ANCOVA: Analysis of covariance
AR: Allergic rhinitis
ARC: Allergic rhinoconjunctivitis
ARTSS: Average rhinoconjunctivitis total symptom score
CI_95%_: 95% confidence interval
DCS: Daily combined score
DRMS: Daily rescue medication score
DRTSS: Daily rhinoconjunctivitis total symptom score
EAACI: European Academy of Allergy and Clinical Immunology
EMA: European Medicines Agency
EudraCT: European Union Drug Regulating Authorities Clinical Trials
FAS: Full analysis set
GINA: Global INitiative for Asthma
IR: Index of reactivity
LS: Least squares
MedDRA: Medical dictionary for regulatory activities
RMS: Rescue medication score
RRTSS: Retrospective rhinoconjunctivitis total symptom score
RSS: Rhinoconjunctivitis symptom score
RTSS: Rhinoconjunctivitis total symptom score
SD: Standard deviation
WAO: World Allergy Organization
